# A Multiplex CRISPR-Screen Identifies PLA2G4A as Prognostic Marker and Druggable Target for HOXA9 and MEIS1 Dependent AML

**DOI:** 10.3390/ijms22179411

**Published:** 2021-08-30

**Authors:** Jacob Jalil Hassan, Anna Lieske, Nicole Dörpmund, Denise Klatt, Dirk Hoffmann, Marc-Jens Kleppa, Olga S. Kustikova, Maike Stahlhut, Adrian Schwarzer, Axel Schambach, Tobias Maetzig

**Affiliations:** 1 Institute of Experimental Hematology, Hannover Medical School, 30625 Hannover, Germany; jacob.j.hassan@stud.mh-hannover.de (J.J.H.); lieske.anna@mh-hannover.de (A.L.); doerpmund.nicole@mh-hannover.de (N.D.); denise_klatt@dfci.harvard.edu (D.K.); dirk.hoffmann@hs-hannover.de (D.H.); kleppa.marc-jens@mh-hannover.de (M.-J.K.); kustikova.olga@mh-hannover.de (O.S.K.); stahlhut.maike@mh-hannover.de (M.S.); schwarzer.adrian@mh-hannover.de (A.S.); schambach.axel@mh-hannover.de (A.S.); 2 Department of Pediatric Hematology and Oncology, Hannover Medical School, 30625 Hannover, Germany; 3 Department of Hematology, Hemostasis, Oncology and Stem Cell Transplantation, Hannover Medical School, 30625 Hannover, Germany; 4 Division of Hematology/Oncology, Boston Children’s Hospital, Harvard Medical School, Boston, MA 02115, USA

**Keywords:** acute myeloid leukemia, leukemic stem cell, *Pla2g4a*, *Hoxa9*, *Meis1*, shRNA, lentiviral vector, multiplexing, fluorescent genetic barcoding

## Abstract

*HOXA9* and *MEIS1* are frequently upregulated in acute myeloid leukemia (AML), including those with MLL-rearrangement. Because of their pivotal role in hemostasis, HOXA9 and MEIS1 appear non-druggable. We, thus, interrogated gene expression data of pre-leukemic (overexpressing *Hoxa9*) and leukemogenic (overexpressing *Hoxa9* and *Meis1*; H9M) murine cell lines to identify cancer vulnerabilities. Through gene expression analysis and gene set enrichment analyses, we compiled a list of 15 candidates for functional validation. Using a novel lentiviral multiplexing approach, we selected and tested highly active sgRNAs to knockout candidate genes by CRISPR/Cas9, and subsequently identified a H9M cell growth dependency on the cytosolic phospholipase A2 (PLA2G4A). Similar results were obtained by shRNA-mediated suppression of *Pla2g4a*. Remarkably, pharmacologic inhibition of PLA2G4A with arachidonyl trifluoromethyl ketone (AACOCF3) accelerated the loss of H9M cells in bulk cultures. Additionally, AACOCF3 treatment of H9M cells reduced colony numbers and colony sizes in methylcellulose. Moreover, AACOCF3 was highly active in human AML with MLL rearrangement, in which *PLA2G4A* was significantly higher expressed than in AML patients without MLL rearrangement, and is sufficient as an independent prognostic marker. Our work, thus, identifies *PLA2G4A* as a prognostic marker and potential therapeutic target for H9M-dependent AML with MLL-rearrangement.

## 1. Introduction

Acute myeloid leukemia (AML) is an aggressive neoplastic disorder characterized by reduced differentiation capacity, increased proliferation, and tissue infiltration of myeloid progenitor cells. While treatment of other malignant hematologic diseases has substantially improved in recent years, prognosis of AML still remains adverse [1]. The challenge to establish long-lasting cures for AML patients lies in the eradication of leukemic stem cells (LSC). Similar to normal hematopoiesis, LSC reside at the apex of the leukemic hierarchy, are self-renewing, and produce immature blasts that trigger most clinical complications. For therapy, disease-specific druggable pathways within shared self-renewal programs of normal and malignant hematopoietic cells must be stringently delineated [2]. For example, the homeobox transcription factor HOXA9 is ultimately required for hematopoietic stem cell (HSC) function, undergoes downregulation with progressing cellular maturation, and increases the self-renewal capacity of HSC upon ectopic expression [3,4,5]. Likewise, the HOXA9 cofactor MEIS1 of the three amino acid loop extension (TALE) family of transcription factors mediates insensitivity towards reactive oxygen species (ROS) and thereby limits HSC exhaustion [6]. In addition, MEIS1 also mediates resistance against differentiation-inducing cues in malignant hematopoiesis [7,8,9]. Although both genes on their own harbor limited transforming potential in mouse studies, their co-expression triggers aggressive disease, which resembles human AML with rearranged MLL (MLLr) [10]. However, due to their pivotal role in normal hematopoiesis, HOXA9 and MEIS1 are considered “non-druggable targets”, making the identification and targeting of leukemia-specific downstream molecules an important medical quest.

*Hoxa9*- and *Meis1*-dependent (H9M) AML can be modelled in mice by the direct overexpression of both transcription factors in bone marrow cells. Transformed cells harbor long-term in vitro expansion capacity while maintaining the potential to trigger aggressive AML in vivo. Since *Hoxa9*-overexpressing cells also grow as stable lines in culture but still respond to differentiation-inducing cues in vitro and in vivo, differential gene and protein expression analyses between Hoxa9 and H9M cells offer the chance to delineate potential oncogenic pathways dispensable for normal hematopoiesis [11]. For example, H9M LSC require signaling through the spleen tyrosine kinase (SYK), which experiences a posttranscriptional increase in protein levels due to Meis1 imposed suppression of miR-146 [12]. Additionally, murine LSC rely on Wnt/β-catenin-dependent gene signatures for the establishment and progression of AML [13,14]. These findings extend to human AML, where β-catenin levels correlate with clonogenic capacity and poor prognosis [14,15], albeit variable responses to pharmacologic intervention have been described [16]. Together, these observations imply that altered signaling cascades in AML offer therapeutic value and may be easier to target than oncogenic lesions.

High-throughput CRISPR/Cas9-mediated knockout screens identified a plethora of potential cancer vulnerabilities [17,18,19,20]. While this approach delivers an unbiased list of potential target genes, these genetic “hits” require validation by independently studying their behavior in focused assays. Conceivably, this approach does not allow for a direct comparison of multiple targets, and, thus, consumes cell culture reagents and time, limiting the number of candidate genes that can be characterized in parallel. To alleviate this obstacle, we and others developed the fluorescent genetic barcoding (FGB) technique [21,22,23,24]. FGB utilizes lentiviral vectors to introduce genetically encoded color codes into dedicated cell samples. After sample mixing, each sample becomes traceable in longitudinal studies through the detection of its color code by flow cytometry. In general, FGB vector systems either make use of a “one color code per vector” approach or utilize combinatorial transductions of differentially color-coded vectors to generate 6 (=6xFGB) to >100 unique fluorescent identifiers [25]. The 6xFGB system appeared most reliable in our hands, since each vector produces a unique color code, optionally accommodates sgRNA expression cassettes, and enables the fate tracking of mixed hematopoietic samples in vitro and in vivo [21].

To close the gap between inefficient small-scale “one gene per sample” and high-throughput sgRNA studies in cancer genomics, we expanded our existing 6xFGB vector platform to up to 24 potential vectors. We used this new 24xFGB platform to functionally validate differentially expressed gene candidates in H9M AML by CRISPR/Cas9 knockout screens. This approach identified PLA2G4A as a new druggable target that may harbor therapeutic and prognostic value for AML patients.

## 2. Results

### 2.1. Identification of H9M-Specific Gene Signatures and Targeting sgRNAs

We analyzed publicly available gene expression data of pre-leukemic *Hoxa9* (H9)- and leukemic H9M-overexpressing murine bone marrow cells with the aim to functionally interrogate the most highly upregulated genes and those that cluster in the leading edge of cancer-associated gene set enrichment analyses (GSEA) [11]. We hypothesized that these genes may harbor prognostic potential, and may additionally serve as molecular targets that warrant further investigation.

**Figure 1:**
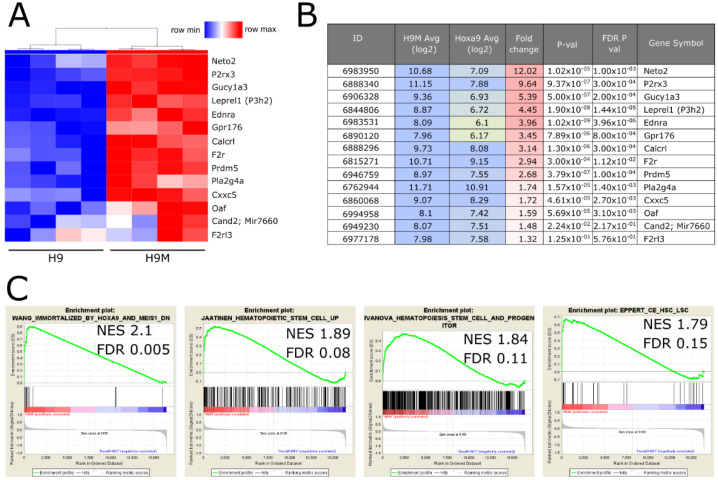
Gene expression analysis of Hoxa9 (H9) vs. Hoxa9/Meis1 (H9M) cells to identify leukemia-essential gene candidates. (**A**) Non-hierarchical clustering of genes selected for functional characterization. (**B**) Summary table of differentially expressed genes between H9M and H9 cells. (**C**) Gene set enrichment analyses. Based on the upregulated genes and those that cluster in the leading edge of the queried gene sets, a candidate gene panel was built for further evaluation.

Gene expression analysis provided a short list of highly upregulated genes in H9M cells (*Neto2* (12.02-fold), *P2rx3* (9.64-fold), *Gucy1a3* (5.39-fold), *Leprel1* (P3h2; 4.45-fold), *Ednra* (3.96-fold), and *Gpr176* (3.45-fold)) that correlated with poor prognosis in The Cancer Genome Atlas (TCGA) dataset (Figure 1A,B) [26].

From the gene sets that had a conservative FDR q-val of 0.25 in GSEA, we more closely investigated upregulated genes that clustered in the leading edge of gene sets “Wang immortalized by Hoxa9 and Meis1 DN” (FDR q-val = 0.009), “Jaatinen Hematopoietic stem cell up” (FDR q-val = 0.102), “Ivanova Hematopoiesis stem cell and progenitor” (FDR q-val = 0.097), and “Eppert CE HSC LSC” (FDR q-val = 0.124) (Figure 1C). From the list of core-enriched genes, upregulated *Prdm5* (2.68-fold), *Cand2* (1.48-fold), *F2rl3* (1.32-fold), *F2r* (2.94-fold), *Calcrl* (3.14-fold), *Cxxc5* (1.72-fold), *Oaf* (1.59-fold), and *Pla2g4a* (1.74-fold) also correlated with poor prognosis in the TCGA data set [26]. Finally, we selected *Itgav* as an additional candidate. Although *Itgav* was not identified in any of our analyses, its heterodimeric cofactor ITGB3 is essential for leukemogenesis and locates upstream of the SYK signaling cascade [27,28]. Together, we curated a list of 15 candidate genes for functional interrogation by a CRISPR/Cas9 mediated knockout approach.

### 2.2. Generation of a Complex Multiplexing Vector Library for Efficient sgRNA Characterization

**Figure 2:**
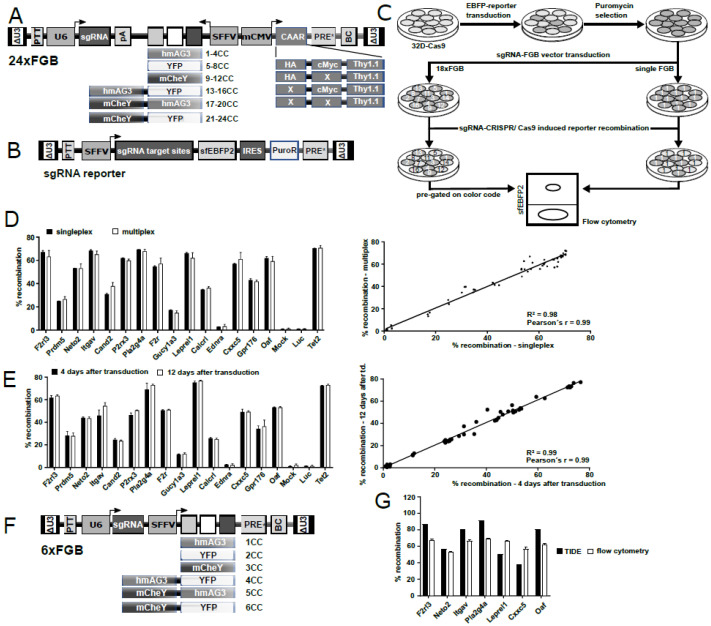
Multiplex sgRNA screen. (**A**) Schematic design of the lentiviral 24xFGB multiplexing vector platform for functional interrogation of sgRNAs. The vector carries a bidirectional design, where a spleen focus-forming virus (SFFV) promoter expresses a fluorescent marker cassette in antisense orientation. The minimal CMV (mCMV) promoter drives the expression of a chimeric antigen array (CAAR) surface marker cassette. The CAAR is made up of a Thy1.1 membrane anchor, which additionally carries permutations of binding and non-binding (X) HA and cMyc epitope tags on its extracellular domain for antibody-mediated detection. The sgRNAs are embedded between the U6 promoter and the inverse polyadenylation signal (pA). (**B**) Schematic design of a fluorescent reporter for the assessment of sgRNA activity. A fusion construct consisting of the translated sgRNA target sites and a superfolder enhanced blue fluorescent protein (sfEBFP2) was cloned between the SFFV promoter and an internal ribosome entry site (IRES)-dependent puromycin resistance gene. (**C**) Schematic experimental design for the assessment of sgRNA activity. 32D cells stably expressing Cas9 were transduced with the sgRNA-sfEBFP2 lentiviral reporter construct (**B**), selected with puromycin and, afterwards, transduced with 18 sgRNA-expressing FGB vectors in independent wells. Transduced cells were assessed for reporter cleavage by flow cytometric analysis of individual wells (singleplex) or of mixes of 18xFGB (multiplex) samples, respectively. (**D**) Comparative recombination efficiency of the sgRNA reporter in singleplex as well as 18xFGB multiplex samples (mean ± SD, *n* = 3) 7 days post transduction. Linear regression analysis revealed a high correlation between singleplex and multiplex measurements (R2 = 0.98, Pearson’s r = 0.99). (**E**) Comparison of recombination rates assessed 4 and 12 days post transduction in singleplex experiments (mean ± SD, *n* = 3). Linear regression analysis showed a stable frequency of recombination between both time points (R2 = 0.99, Pearson’s r = 0.99). (**F**) Schematic vector design of the 6xFGB platform for the assessment of sgRNA activity. The U6 promoter drives the sgRNA expression. Likewise, the SFFV promoter drives the expression of a fluorescent marker cassette, and each of the color codes (CC) carries a unique DNA barcode (BC). (**G**) Comparison of gene editing efficiencies in 32D cells measured by flow cytometry versus H9M-Cas9 cells based on TIDE analysis (mean ± SD, TIDE n = 1, flow cytometry *n* = 3). ∆U3, self-inactivating long terminal repeat; PPT, polypurine tract; hmAG3, human codon-optimized Azami Green fluorescent protein variant; YFP, yellow fluorescent protein; mChEY, monomeric mCherry fluorescent protein variant; PRE*, posttranscriptional regulatory element; BC, DNA barcode.

To efficiently select potent sgRNAs against the 15 candidate genes, we first increased the complexity of our 6xFGB multiplexing vector platform to produce up to 24 different color codes. This was accomplished by using a bidirectional vector configuration. Here, the six fluorescent marker combinations from the 6xFGB vector platform were placed under control of the spleen focus forming virus (SFFV) promoter in antisense orientation. In addition, a novel chimeric antigen array (CAAR) consisting of a Thy1.1 membrane anchor equipped with and without hemagglutinin (HA) and cMyc epitope tags was expressed from a minimal CMV promoter in sense orientation. A combination of the six fluorescent marker cassettes with our four new CAAR cassettes generated a total of 24 vector-specific color codes (Figure 2A). For multiplexing experiments, each vector would need to be transduced into a separate cell sample, prior to sample mixing for longitudinal tracking by flow cytometric deconvolution (Figure S1A). Additionally, each vector could also be characterized in singleplex experiments for functional validation.

SgRNAs against all 15 candidates were selected by CCTop online tool [29] and cloned into the lentiviral 24xFGB vector system along with three putative controls (Tet2, Luciferase and empty control) (Figure 2A). By making use of our new 24xFGB vector platform, each sgRNA was linked to a specific color code traceable by flow cytometry. The cleavage efficiency of all sgRNAs was subsequently tested in a reporter assay that utilized a lentivirally integrated array of sgRNA target sites in the 5′region of a super folder EBFP2 (sfEBFP2) within Cas9-overexpressing promyelocytic murine 32D cells (Figure 2B,C) [30]. In flow cytometric singleplex and multiplex assays with 18 FGB vectors (15 candidate genes and three controls), cleavage efficiencies were calculated by the percentage of EBFP2 positive and negative cells in each color-coded population. These experiments revealed cutting efficiencies of >50% for sgRNAs against *F2rl3* (66.7%), *Neto2* (52.2%), *Itgav* (66.1%), *P2rx3* (61.3%), *Pla2g4a* (69.8%), *F2r* (56.6%), *Leprel1* (69.9%), *Cxxc5* (56.7%), and *Oaf* (63.6%), as well as for the Tet2 (70.9%) positive control (Figure 2D) and a high correlation between singleplex and multiplex determined recombination rates (Pearson’s r = 0.99, R2 = 0.99) regardless of the requirement to stain for surface CAAR epitopes (Figure 2D). Notably, stable recombination rates were detected as early as 4 days after transduction (Pearson’s r = 0.994, R2 = 0.9876, Figure 2E). This demonstrates the applicability of our improved multiplexing approach for forthcoming sgRNA knockout experiments and longitudinal tracing of knockout cells.

Taken together, we identified highly active sgRNAs against 9 of our 15 target genes for subsequent functional characterization, and established a reliable approach for the parallel investigation of sgRNA cleavage efficiencies and possibly cell fate consequences by flow cytometry.

### 2.3. sgRNAs Identified in 32D-Cas9 Reporter Assays Are also Highly Active in H9M Cells

We next attempted to investigate the functional implications of target gene knockout on the growth behavior of H9M cells derived from Cas9-expressing bone marrow cells (H9M-Cas9). However, transductions of the sgRNA-expressing 18xFGB vector library into H9M-Cas9 cells resulted in only very low gene transfer rates (<12.5%), presumably due to low bidirectional vector titers combined with the resistance of H9M cells against lentiviral integration (Figure S2A,B) [31]. Therefore, we cloned the selected 9 sgRNAs into our established unidirectional 6xFGB vectors, and also included Gpr176 as the 10th most active sgRNA into the panel. This enabled the production of two 6xFGB vector sets, each consisting of five sgRNAs and one empty control (Figure 2F), which could be concentrated to high titers and facilitated gene transfer rates between 15–65% in H9M-Cas9 cells (Figure S2A,B). Due to the absence of an integrated fluorescent sgRNA reporter construct in these cells, we assessed the cleavage efficiency within PCR amplified target sequences and subsequent deconvolution of Sanger sequencing chromatograms using the Tracking of Indels by DEcomposition (TIDE) analysis online tool [32]. When normalizing data by gene transfer rates, cleavage efficiencies of all sgRNAs reached ≥50% except for *Cxxc5* (38%, Figure 2G). Notably, TIDE did not provide meaningful data for *P2rx3*, *F2r* and *Gpr176* suggesting that computational analyses occasionally need to be complemented by alternative approaches.

These data demonstrate that sgRNA cleavage efficiencies determined by fluorescent reporter assay in 32D-Cas9 cells and by computational analysis in H9M-Cas9 cells correlate well, even though slightly different (6xFGB vs. 18xFGB) vector constructs were used.

### 2.4. Competition Assays Identify Pla2g4a as a Putative Target for Cancer Therapy

**Figure 3:**
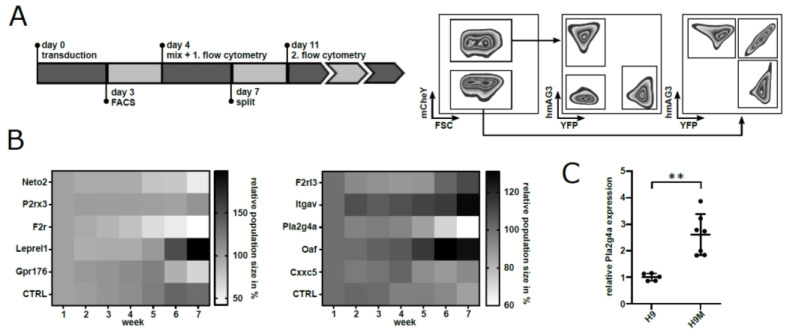
sgRNA screen in H9M cells reveals a growth requirement for PLA2G4A. (**A**) Experimental timeline. After purification of gene marked sgRNA-expressing cells, cell mixes of six different samples (5 sgRNAs + 1 control) were created and subsequently analyzed by flow cytometry for population sizes. A representative gating strategy for the assessment of population sizes is shown on the right. (**B**) Heatmap representing relative population sizes of sgRNA-transduced H9M cells in 6xFGB mixes over time (*n* = 3). After 7 weeks, the *Pla2g4a* knockout population showed a decreased median population size of 63.68% compared to the initial measurement, while the control remained stable at 91.23%. (**C**) RT-qPCR analysis of *Pla2g4a* expression of independently transduced Hoxa9 (H9) and Hoxa9/Meis1 (H9M) cell lines (scatter dot plot, mean ± SD). *Pla2g4a* was significantly upregulated in H9M cells (Student’s *t*-test, *p* = 0.0011, mean = 2.61; ** = *p* < 0.005).

To investigate the functional consequences of target gene knockout, we subsequently designed two separate competition assays consisting of five FGB vectors expressing sgRNAs against our candidate genes as well as one FGB vector without sgRNA as a negative control. After stoichiometric combination of all six vector-transduced H9M-Cas9 samples, competitive growth behavior of sgRNA-modified cells was tracked via fluorescent marker expression (Figure 3A and Figure S1B). In two independent experiments, multiplex assays reported a loss of color codes linked to *Pla2g4a* and *F2r* targeting sgRNAs in comparison to controls (Figure 3B). Due to the 2.6-fold upregulation of *Pla2g4a* mRNA in H9M in comparison to H9 cells, as determined by quantitative RT-qPCR (Figure 3C), we next opted to further validate *Pla2g4a* importance for H9M growth by independent techniques.

### 2.5. shRNA-Mediated Knockdown of Pla2g4a Recapitulates sgRNA-Induced H9M Growth Delay

**Figure 4:**
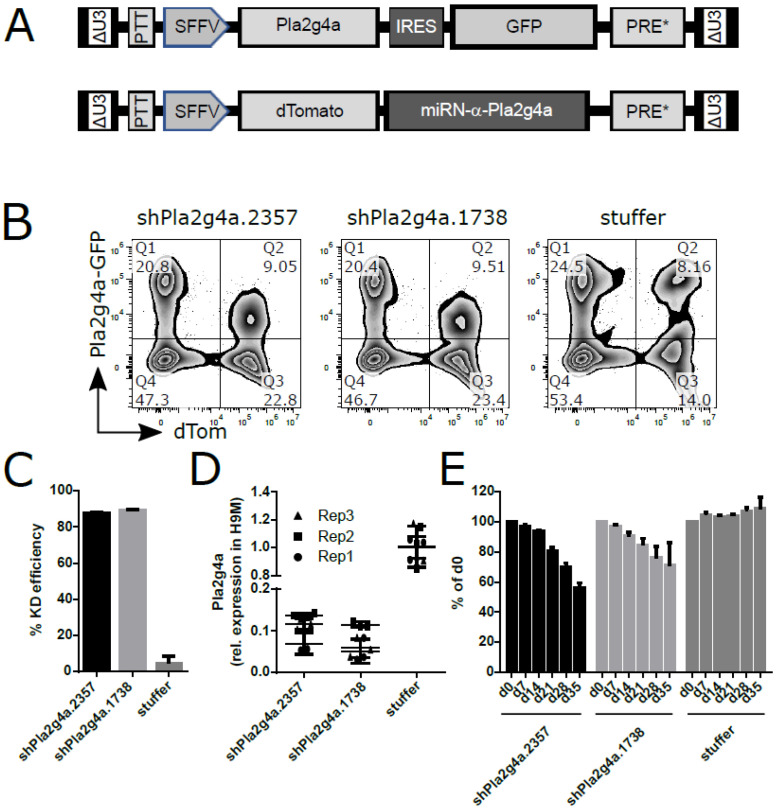
*Pla2g4a* knockdown impairs H9M cell growth. (**A**) Schematic representation of (top) a lentiviral vector co-expressing *Pla2g4* and GFP via an internal ribosome entry site (IRES) and (bottom) an shRNA expression vector co-expressing dTomato and a mir-N embedded hairpin against *Pla2g4a* under control of the SFFV promoter. (**B**) Representative flow cytometric analysis plots of two shRNAs against *Pla2g4a*, which downregulate the signal of a PLA2G4A-GFP reporter. Transduction with an empty shRNA vector (stuffer) does not cause loss of *Pla2g4a* reporter expression. (**C**) Calculation of the knockdown efficiency of two shRNAs as well as of the “stuffer” control vector based on data from the reporter assay from (**B**). (**D**) Validation of *Pla2g4a* knockdown efficiency by RT-qPCR in three different H9M lines (Rep1, Rep2, and Rep3), in comparison to a non-targeting (stuffer) miR-N backbone. (**E**) Depletion of shRNA-α-*Pla2g4a* transduced H9M cells over time. Data shown from one exemplary experiment with three data points per vector. Data were normalized to the gene marking rate at the first measurement (d0) seven days after the initial transduction.

Two separate miR-N framework-embedded shRNAs against *Pla2g4a* were cloned into a lentiviral dTomato co-expressing vector (Figure 4A) [33]. In addition, the control vector was cloned without a targeting hairpin. In a fluorescent reporter assay, both shRNAs reduced Pla2g4a signals by 88% and 89%, respectively, and these results were confirmed by RT-qPCR of transduced H9M cells (Figure 4B–D). Subsequent growth assays revealed the depletion of shRNA-α-*Pla2g4a*-expressing H9M cells over time (Figure 4E). Importantly, the kinetics of growth delay correlated to sgRNA-mediated suppression of *Pla2g4a* (Figure 3B).

In summary, our results indicate that molecular inhibition of *Pla2g4a* impairs population growth of H9M cells and plays an important role in cellular fitness.

### 2.6. Drug-Inhibition of PLA2G4A Impairs H9M Cell Growth

**Figure 5:**
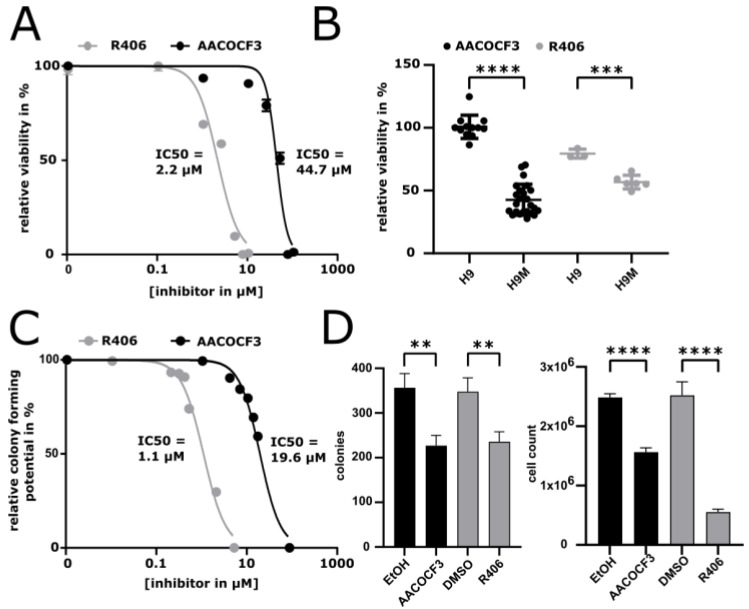
Pharmacologic inhibition of PLA2G4A specifically impairs H9M cell growth. (**A**) Determination of the IC50 of the PLA2G4A inhibitor AACOCF3 (44.7 µM) and the SYK inhibitor R406 (2.2 µM) based upon viability of H9M cells determined with the resazurin-based alamarBlue assay. Viability was normalized to H9M cells treated with DMSO or EtOH as a vehicle control (dose response curve, *n* = 7). (**B**) alamarBlue viability assay of H9M and H9 cells after 24 h exposure with AACOCF3 (44.7 µM) and R406 (2.2 µM), respectively. Viability was normalized to H9M/ H9 cells treated with a vehicle control (DMSO/ EtOH). Both inhibitors showed a significant loss of viability of H9M cells compared to H9 cells (Scatter dot plot, Student’s *t*-test, mean ± SD: AACOCF: mean relative viability H9 = 100.8% (*n* = 12), H9M = 42.66% (*n* =24), *p* = <0.0001; R406: H9 = 79.44% (*n* = 3), H9M = 56.74% (*n* = 6), *p* = 0.0003). (**C**) Determination of the colony-forming potential (CFP) IC_50_ of AACOCF3 (19.6 µM) and R406 (1.1 µM) in H9M cells using the methylcellulose colony-forming assay (CFA). Colony-forming potential was normalized to H9M cells treated with DMSO or EtOH as a vehicle control (dose response curve). (**D**) 500 H9M cells were seeded in a CFA with R406 (1.1 µM), AACOCF3 (19.6 µM) or a vehicle control (DMSO/ EtOH). Experiments were performed in duplicates of three independently generated H9M lines. Both inhibitors caused a significant reduction in colonies (mean ± SD, *n* = 3, Student’s *t*-test: AACOCF3: *p* = 0.0045, R406: *p*= 0.0071) and absolute cell numbers (mean ± SD, *n* = 3, Student’s *t*-test, AACOCF3: *p* < 0.0001, R406: *p* = 0.0001) compared to the vehicle control. ** = *p* < 0.005; *** = *p* < 0.0005; **** = *p* < 0.0001.

As chemical inhibitors currently provide greater applicability for clinical translation than molecular interventions, we investigated the potency of arachidonyl trifluoromethyl ketone (AACOCF3) to interfere with H9M cell growth. AACOCF3 represents an arachidonic acid (AA) analog commonly used to specifically inhibit PLA2G4A [34,35,36]. Notably, AACOCF3 interfered with H9M cell growth and the IC_50_ was determined to be 44.7 μM (95% CI = 39–52 μM) in short-term alamarBlue growth assays (Figure 5A). This is similar to the range of AACOCF3 concentrations used in previous studies [34]. Importantly, exposure of Hoxa9-expressing BM cells to the same AACOCF3 concentration resulted in only marginal growth reduction as compared to the control, implying that PLA2G4A inhibition may specifically interfere with leukemogenic pathways (Figure 5B). In contrast, the SYK inhibitor R406, previously described as an effective small molecule to inhibit H9M-driven leukemogenesis [12], showed a stronger impairment of H9 cells (Figure 5A,B). Therefore, AACOCF3 may exhibit a higher specificity for leukemic cells than R406 (Figure 5B). Since these short-term assays only assess the growth potential of bulk cultures, we next performed colony-forming cell (CFC) assays with H9M cells to better reflect the growth potential of immature self-renewing cells. Due to the longer exposure of H9M cells to AACOCF3 when cultured in methylcellulose, the IC_50_ was already reached at 20 μM (Figure 5C). A similar reduction in IC_50_ was also found for R406 (Figure 5C). Regardless, H9M cells exposed to IC_50_ levels of AACOCF3 during 7 days of cultivation in methylcellulose resulted in a 36.3% reduction in colony formation and 37.1% of cells therein in comparison to untreated control cells (Figure 5D). Interestingly, while R406-treated H9M cells also caused a 32.1% depletion of colonies, these colonies contained 78% less cells compared to the controls (Figure 5D). Together, this shows that chemical PLA2G4A inhibition mediates a comparable reduction in the frequency of self-renewing cells as SYK inhibition.

### 2.7. PLA2G4A Inhibition Interferes with the Growth of Human AML Cells

**Figure 6:**
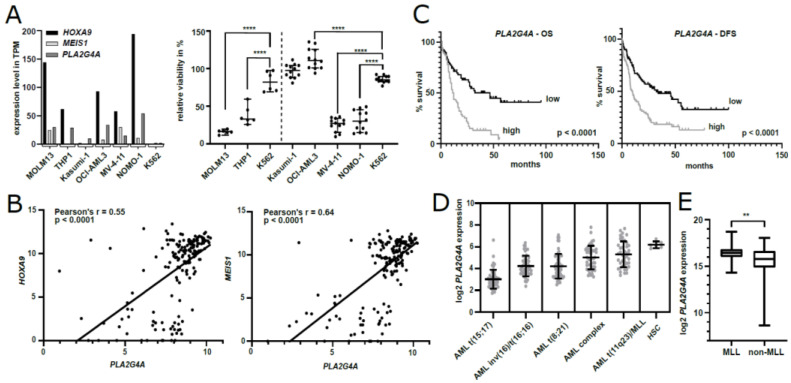
Characterization of PLA2G4A in human AML. (**A**) Left: expression levels of *HOXA9*, *MEIS1*, and *PLA2G4A* within MOLM13, THP1, Kasumi-1, OCI-AML3, MV-4-11, NOMO-1, and K562 cells based on RNA sequencing data from the Cancer Cell Line Repository. Right: alamarBlue viability assay of MOLM13, THP1, Kasumi-1, OCI-AML3, MV-4-11, NOMO-1, and K562 cells after 24 h exposure to 45 µM AACOCF3 normalized to vehicle control-treated cells. The AML MLLr cell lines (MOLM13, MLL-AF9; THP1, MLL-AF9; MV4-11, MLL-AF4; and NOMO-1, MLL-AF9) showed a significant reduction in viability compared to the control K562 blast phase chronic myeloid leukemia cell line. Unrelated AML lines OCI-AML3 (wt. TP53 and NPM1c) and Kasumi-1 ((RUNX1/AML1-RUNX1T1/ETO) were not negatively impacted by the treatment. ****, *p* < 0.0001 with one-way ANOVA, Dunnett’s multiple comparison test. (**B**) Co-expression of *PLA2G4A* with *HOXA9* (left) and *MEIS1* (right), respectively, in AML samples from the TCGA-LAML data set. *PLA2G4A* expression significantly correlates with *HOXA9* (*p* < 0.0001, Pearson‘s r = 0.55) and *MEIS1* (*p* < 0.0001, Pearson‘s r = 0.64) (linear regression analysis). (**C**) Kaplan–Meier survival curve of overall survival (OS) and disease-free survival (DFS) in AML patients with above median *PLA2G4A* expression (*n* = 61) compared to patients with below median *PLA2G4A* expression (*n* = 60). The high *PLA2G4A* expression group had significantly lower OS (Mantel–Cox test: *p* < 0.0001, median OS = 12.2 months vs. 46.7 months) and DFS (Mantel–Cox test: *p* < 0.0001, median DFS = 9.6 vs. 32.3 months) compared to the control group. (**D**) *PLA2G4A* gene expression in patient samples with different AML subtypes and HSC of healthy individuals (scatter dot plot, mean ± SD: mean log2 *PLA2G4A* expression: AML t(15;17) = 3.0, AML inv[1 6]/t(16;16) = 4.2, AML t(8;21) = 4.2, AML complex = 5.0, AML t(11q23)/MLL = 5.3, HSC = 6.2). (**E**) Box plot of *PLA2G4A* expression in MLLr or non MLLr AML patient samples. The upper, center, and lower limit of each box denotes the upper quartile, median, and lower quartile, respectively. Whiskers representing min and max expression samples. *PLA2G4A* is significantly upregulated in AML patient samples with MLLr (*n* = 30) compared to those without (*n* = 142) (Welch‘s *t*-test: *p* = 0.0015, mean log2 PLA2G4A expression: MLL-AML = 16.34, non-MLL-AML = 15.67. TPM, transcripts per kilobase million. ** = *p* < 0.005; **** = *p* < 0.0001.

To investigate the potency of PLA2G4A inhibition to treat human AML, representative MLL-AF9 recombined cell lines THP1 and MOLM13 as well as the blast phase chronic myeloid leukemia (CML) K562 control cell line were treated with IC_50_ levels of AACOCF3 previously determined on H9M cells. These lines were selected based on their differential expression of *HOXA9*, *MEIS1*, and *PLA2G4A* in the Cancer Cell Line Repository sequencing data (Figure 6A) [37,38]. In alamarBlue growth assays, both AML cell lines showed a drastic decrease in cell viability compared to the vehicle control (median relative viability: THP1 = 32.9%, MOLM13 = 16.4%), while AACOCF3 exposure of CML cells only caused a modest growth impairment (K562 = 83%, Figure 6A). Next, we investigated the effect of PLA2G4A inhibition on additional MLLr AML lines (Nomo-1 (MLL-AF9) and MV-4-11 (MLL-AF4)) as well as of unrelated AML lines (Kasumi-1 (RUNX1/AML1-RUNX1T1/ETO) and OCI-AML3 (NPM1c)). Notably, OCI-AML3 cells harbor the NPM1c mutation and, thus, demonstrate *HOXA9* upregulation independent from MLL. Again, treatment with AACOCF3 significantly depleted MLLr cells, while the other AML lines and the K562 control cells remained viable. This suggests that PLA2G4A inhibition may be especially effective against AML with MLLr or direct *HOXA9* and *MEIS1* upregulation.

### 2.8. PLA2G4A Serves as a Prognostic Marker in Human AML

Initially, we found the *Pla2g4a* candidate gene to be upregulated in murine H9M cells in comparison to pre-leukemic H9 cells. Interestingly, *PLA2G4A* expression also correlates with *HOXA9* and *MEIS1* expression in human AML (Figure 6B). Thus, we interrogated the TCGA data set for a potential correlation between *PLA2G4A* expression level and survival. In accordance with recently published studies, *PLA2G4A* correlated with unfavorable prognosis [39,40]. We used median *PLA2G4A* gene expression for stratification, which demonstrated significantly poorer prognosis regarding disease-free survival (9.6 vs. 32.2 months; *p* < 0.0001) and overall survival (12.2 vs. 46.7 months; *p* < 0.0001) in patients with high *PLA2G4A* expression compared to individuals with low *PLA2G4A* expression (Figure 6C). Moreover, we compared *PLA2G4A* expression levels between healthy and malignant hematopoiesis using the bloodspot normal hematopoiesis with AMLs sample set [41]. This showed the highest *PLA2G4A* levels in AML with t(11q23)/MLL, albeit at lower levels than in healthy hematopoietic stem cells (Figure 6D). Notably, progenitor populations expressed *PLA2G4A* at least at similar levels as observed in HSC (data not shown). In addition, *PLA2G4A* levels were significantly upregulated in pediatric patients with MLL fusions compared to wild-type MLL patients in the TARGET AML data set (*p* = 0.0015) (Figure 6E).

Collectively, our data indicate that *PLA2G4A* expression may hold therapeutic and prognostic potential for the stratification of AML patients and might play a special role in subtypes with MLL-fusions and/or elevated *HOXA9* and *MEIS1* levels.

## 3. Discussion

In this work, we established the use of our FGB system in combination with CRISPR/Cas9 as an efficient tool to identify gene dependencies of leukemic cells in multiplex assays. This approach led to the identification of PLA2G4A as a potential target and prognostic marker for H9M-driven leukemia.

PLA2G4A belongs to the group IV phospholipase A2 family (cPLA2) that hydrolyses phospholipids for the provision of AA as the rate-limiting substrate for prostanoid production. Prostaglandin endoperoxide synthase (PTGS; aka cyclo-oxygenase (COX)) subsequently converts AA into a prostaglandin (PG) H_2_ intermediate, which is then further processed by prostaglandin E synthase (PTGES) into PGE_2_ as well as into thromboxanes, prostacyclins, and additional prostaglandins by alternative tissue specific isomerases [42]. Typically, prostaglandins function as potent immune modulators, but also play important roles for tumor initiation, promotion, invasion, and metastasis [42,43,44,45,46,47].

Initially, we hypothesized that upregulated genes and genes that cluster in the leading edge of leukemia-associated gene sets may harbor leukemia-essential properties and prognostic value (Figure 1). To efficiently test our 15 candidates from the comparison between leukemic H9M and pre-leukemic H9 cells, we first established a multiplexing approach that enabled the identification highly active sgRNAs for nine target genes in reporter assays, and validated these results on the genomic level by locus amplification and TIDE analysis (Figure 2). Regardless, only *F2r* and *Pla2g4a* knockouts caused H9M cell depletion in longitudinal multiplex assays (Figure 3), suggesting that the MEIS1-dependent upregulation of at least some candidate genes represents passive events [11]. Interestingly, F2R and PLA2G4A both impinge on β-catenin, as F2R (aka proteinase-activated receptor 1 (PAR1)) potentiates Wnt signaling and concomitantly suppresses JNK [48,49], while PLA2G4A locates upstream of PGE_2_ receptor (PTGER)-signaling and β-catenin-dependent TCF/LEF target gene transcription [13,42,50]. Since the importance of PGE_2_-mediated induction of β-catenin for the formation and maintenance of LSC was initially discovered through upregulation of *Ptger* and *Ptgs1* in murine leukemia [13], we subsequently focused on the investigation of *Pla2g4a* as a leukemia essential gene. Interestingly, MEIS1 binding to the *Pla2g4a* locus has previously been observed in ChIP-Seq data [51]. Although only weakly upregulated in H9M cells (1.74-fold by microarray and 2.6-fold by RT-qPCR; Figure 1A,B and Figure 3C), it essentiality could be further validated by shRNA-mediated depletion and pharmacologic inhibition (Figure 4 and Figure 5). However, in this context it remains unclear, why AACOCF3 treatment caused significantly faster cell depletion than sgRNA- and shRNA-mediated approaches (Figure 3, Figure 4 and Figure 5). We hypothesize that AACOCF3 also inhibits other targets that contribute to the same signaling loop as PLA2G4A. As such, PTGS2 (aka COX-2) appears transcriptionally co-regulated with PLA2G4A, constitutes a β-catenin target gene, and may occasionally also be targeted by AACOCF3 [34,52,53]. This may explain the stronger phenotype seen with chemical (putative PLA2G4A and PTGSs) inhibition than by targeted molecular reduction in PLA2G4A levels. An additional explanation depends on prostanoid’s mode of action: since the sgRNA and shRNA experiments were conducted in multiplex assays, a considerable amount of cells within the cultures did not exhibit PLA2G4A reductions and, thus, presumably still secreted paracrine-acting prostanoids, potentially delaying growth interference through cis-acting PLA2G4A suppression.

Regardless, PLA2G4A embeds into a well-described oncogenic signaling cascade with multiple druggable targets, such as MAP kinases, the tyrosine-protein kinase SYK, and the prostaglandin G/H synthase 2 (PTGS2 = COX-2), respectively (Figure S3) [12,13,50,54]. Our work now suggests that also directly targeting PLA2G4A may hold therapeutic potential, as demonstrated by the comparable growth-delaying effect of AACOCF3 on murine H9M cells as well as on human AML lines with recombined MLL (Figure 5 and Figure 6A). Notably, despite functional *TP53*, OCI-AML3 cells did not respond to AACOCF3 treatment.

Because of the relatively high IC_50_ of AACOCF3 (~45µM), for translational studies, alternative PLA2G4A inhibitors with better oral activity, better bioavailability, and lower IC_50_—such as ASB14780—may be preferable [55]. During 16 weeks of treatment, mice did not show hematologic complications, suggesting that even systemic PLA2G4A inhibition does not cause overt toxicity [56]. Therefore, it would be interesting to also investigate possible synergistic effects of PLA2G4A inhibitors with drugs indirectly interfering with the activation and/or maintenance of HOXA9- and MEIS1-dependent transcriptional programs, for example, through MENIN inhibition, DOT1L inhibition, and nuclear export inhibition [57,58,59].

Despite the initial observation of upregulated PGE_2_ pathway components in murine leukemia [13], we could not associate prognostic value to PTGS, PTGES, and PTGER in human AML (Figure S4). Instead, only increased *PLA2G4A* expression predicted adverse outcome (Figure 6B), which was also independently confirmed [39,40]. This is in line with the already described oncogenic properties of PLA2G4A in various cancers [34,60,61,62], and supports the functional and prognostic value of PLA2G4A in AML that warrants further investigations.

## 4. Materials and Methods

### 4.1. Viral Vectors

Retroviral LTR-driven vectors pRSF91.Hoxa9.i2.Puro.LVpre and pRSF91.Meis1-2A-Hoxa9.i2.Puro.LVpre contained the murine cDNAs of *Meis1* and/or *Hoxa9* followed by an internal ribosome entry site (i2) and the puromycin N acetyltransferase genes (puro) for chemical selection of transduced cells. The previously described unidirectional 6xFGB vector design was slightly modified by exchanging the eGFP and mKO3 coding sequences for modified versions of the monomeric Azami Green and mCherry fluorescent proteins for ease of cloning and flow cytometric detection [63]. Bidirectional FGB vectors were cloned by placing a surface marker cassette consisting of permutations of Thy1, cMyc-tag, and HA-tag under the control of a minimal cytomegalovirus (mCMV) promoter fused to the inverted SFFV-xFP cassette from unidirectional FGB vectors [31]. Each of these vectors carries a unique DNA barcode for molecular detection. Cloning details are available on request. Notably, all FGB vectors contain a 2xBbsI linker in their 5′ leader region to facilitate the incorporation of sgRNA expression units by golden gate assembly (see below). The lentiviral sgRNA reporter vector (pRRL.PPT.Cbx3-SFFV.Rep.sfEBFP2.i2.Puro.pre*) was generated by first stringing together all designated sgRNA target sites (sgRNA sequence plus 3bp PAM motive) while removing potential in-frame stop codons through the introduction of random nucleotides between the different target sites. This reporter array was ordered as a synthesized DNA fragment, subcloned, and sequence-verified before insertion into the 5′region of an EBFP2 cDNA equipped with super-folder mutations and located 5′ of an ires.puro selection cassette.

### 4.2. Cell Culture

293T cells were cultivated in DMEM^+++^, consisting of Dulbecco’s Modified Eagle Medium (Biochrom, Berlin, Germany) supplemented with 10% heat-inactivated (h.i.) fetal bovine serum (FBS; Brazil One or FBS Standard, PanBiotech, Aidenach, Germany), 100 U/mL of penicillin, 0.1 mg/mL of streptomycin (1% PS), and 1 mM of sodium pyruvate (both PanBiotech, Aidenach, Germany). Cells were split by trypsin (PanBiotech, Aidenach, Germany) digestion. 32D and 32D-Cas9 cells were incubated in RPMI 1640 (PanBiotech, Aidenach, Germany) supplemented with 10% h.i. FBS, 1% PS, and 2 ng/mL of murine interleukin-3 (mIL3; Peprotech, Hamburg, Germany). MOLM13, THP1, K562, MV-4-11, and NOMO-1 were cultivated in RPMI 1640 supplemented with 10% h.i. FBS and 1% PS. Kasumi-1 were cultivated in RPMI 1640 supplemented with 20% h.i. FBS and 1% PS. OCI-AML3 cells were cultivated in alpha-MEM (with ribo- and deoxyribonucleosides; Gibco/ThermoFisher Scientific, Waltham, MA, USA) supplemented with 20% h.i. FBS and 1% PS. Murine lineage-depleted bone marrow cells, Hoxa9 cells, and Hoxa9/Meis1 cells were cultivated in 36SF medium consisting of Dulbecco’s Modified Eagle Medium (Biochrom, Berlin, Germany) supplemented with 15% h.i. FBS, 1% PS, and 1 mM sodium pyruvate (PanBiotech, Aidenach, Germany), as well as 6 ng/mL of mIL3, 10 ng/mL of human interleukin-6, and 20–100 ng/mL of murine stem cell factor (mSCF). All cytokines from Peprotech, Hamburg, Germany. All cells were incubated at 37 °C and 5% CO_2_ in a humidified incubator.

### 4.3. Generation of 32D-Cas9, H9 and H9M Cell Lines

32D-Cas9 cells were generated by transduction of 32D cells with the lentiviral vector pLKO5d.EF1a.hSpCas9-P2A-BSD.pre encoding for Cas9 and the blasticidin resistance (Bsd) gene followed by selection with 10 µg/mL of blasticidin for one week. For the generation of Hoxa9-Cas9 and Hoxa9/Meis1-Cas9 cells, bone marrow was harvested from B6J.129(Cg)-Gt(ROSA)26Sor^tm1.1(CAG-cas9*,-EGFP)Fezh/J^ mice and subjected to lineage depletion using the MojoSort Mouse Hematopoietic Progenitor Cell Kit (BioLegend, San Diego, CA, USA), according to the manufacturer’s instructions. Lineage-depleted cells were pre-stimulated for two days in 36SF medium prior to seeding of 5 × 10^4^ cells per 96 U-bottom well in 36SF medium supplemented with 4 µg/mL of protamine sulfate for transduction with concentrated VSVg pseudotyped gammaretroviral supernatants (pRSF91.Hoxa9.i2.Puro.LVpre and pRSF91.Meis1-2A-Hoxa9.i2.Puro.LVpre). Two days later, cells were transferred into 36SF medium supplemented with 1 µg/mL of puromycin and expanded for at least 7 days. Afterwards, aliquots of cells were frozen and thawed as needed.

### 4.4. Vector Production and Transduction

Lentiviral and retroviral vectors were produced by transient transfection of 293T cells by calcium phosphate method according to standard protocols. In brief, 6 × 10^6^ 293T cells were seeded in 10-cm plates the day before transfection. Prior to transfection, the medium was exchanged with DMEM^+++^ supplemented with 20 mM of HEPES (PanBiotech, Aidenach, Germany) and 25 µM of chloroquine (Sigma Aldrich, Steinheim, Germany). Transfections were carried out with 7 µg of lentiviral backbone plasmid, 6 µg of pRSV-Rev, 9 µg of pcDNA3.g/p.4xCTE, and 2 µg of pMD.G (VSVg). For bidirectional vectors, 5 µg of pcDNA3.NovB2 was additionally added to increase titers [31]. Production of retroviral vectors was carried out with 7 µg of retroviral backbone plasmid, 6 µg of pcDNA3.MLVg/p, and 1.5 µg of pMD.G. Sixteen hours post transfection, the cells received fresh DMEM^+++^ medium supplemented with 20 mM of HEPES, and supernatants were collected 24 and 48 h later. Supernatants were subsequently pooled and filtered (0.22 µm) prior to concentration by ultracentrifugation for 2 h at 4 °C in a SW32Ti rotor at 25,000 rpm. Viral pellets were resuspended in a small volume and frozen at −80 °C for later use.

### 4.5. alamarBlue Cell Viability Assay

5 × 10^4^–1 × 10^5^ cells were seeded in a 96-well flat-bottom plate as quadruplicates with the respective small molecule concentrations or DMSO/ EtOH as a vehicle control. After 20–24 h of incubation (37 °C and 5% CO_2_) alamarBlue (ThermoFisher Scientific, Waltham, MA) was added to assess cell viability. Absorbance was measured 4 h after the addition of the alamarBlue reagent at a wavelength of 570/600 nm using a Benchmark Plus microplate reader (Bio-Rad, Hercules, CA, USA). Medium alone was used as a blank. Statistical analysis and IC_50_ values were calculated using Graphpad Prism 8 (GraphPad Inc., San Diego, CA, USA).

### 4.6. Colony-Forming Assay

200 cells were seeded in 450 µL MethoCult GF M3434 (STEMCELL Technologies, Vancouver, BC Canada) as duplicates with the respective small molecule concentrations or DMSO/EtOH as a vehicle control. After 7 d of incubation at 37 °C and 5% CO_2_, colonies were counted. For cell number determination, cells were eluted from methylcellulose, washed and then counted. Statistical analysis and IC_50_ values were calculated using Graphpad Prism 8 (GraphPad Inc., San Diego, CA, USA).

### 4.7. Design and Cloning of Targeting Constructs (shRNA and sgRNA)

shRNAs design and cloning into the lentiviral miR-N framework followed the strategy described by Adams et al. [33]. Oligonucleotide sequences against *Pla2g4a* are summarized in Supplementary Table S1. sgRNAs were designed using the CCTop online tool [29], and ordered as two complementary oligonucleotides with golden gate assembly compatible overhangs for incorporation into FGB vectors. In brief, the FGB vector backbone was mixed with assembled sgRNA oligos, a double-stranded linker and a plasmid providing the hU6 promoter as well as the sgRNA-derived tracrRNA framework for sequential digestion (37 °C) and ligation (16 °C) with the type IIS restriction enzyme BbsI and T4 DNA ligase, respectively. After bacterial transformation, the newly assembled plasmids were control digested and verified by sanger sequencing. Target sites and oligonucleotide sequences for cloning are depicted in Supplementary Table S2.

### 4.8. Flow Cytometry

Cells were gathered and washed in staining buffer containing 1% FBS and 2 mM of EDTA in PBS. In experiments utilizing the 24xFGB vector system, cells were first incubated with a biotinylated anti-cMyc antibody (Miltenyi Biotec, Bergisch Gladbach, Germany) for 30 min at 4 °C, and, after washing, they were subsequently stained for 30 min in the dark at 4 °C with an antibody cocktail consisting of streptavidin-conjugated APC secondary antibodies, Thy1.1-PE-Cy7 (clone OX-7) (both Biolegend) and HA-PE (Miltenyi, Bergisch Gladbach, Germany). Prior to analysis, cells were washed and resuspended in staining buffer containing 0.1 µg/mL of DAPI for live/dead cell exclusion. No prior antibody staining was needed for experiments using the 6xFGB vector system. Data were acquired with the CytoFlex S (Beckman Coulter, Brea, CA, USA), FACSCalibur, or FACSCanto (both BD, Franklin Lakes, NJ, USA). For cell sorting, the BD FACSAria Fusion cell sorter was used. Data analysis was performed with FlowJo 10 (BD, Franklin Lakes, NJ, USA). Gating strategies are displayed in Supplementary Figure S1.

### 4.9. Competitive Proliferation Assay

1 × 10^5^ H9M cells were transduced with 6xFGB-sgRNA vectors. Three days after expansion, the transduced cells were sorted and separated from the non-transduced cells. The cells were expanded for one day. Then, ~1000 transduced cells of each color code were mixed. An aliquot of the mixture was analyzed immediately by flow cytometry. After three days, the remaining cells from each mixture were split into three separate wells, allowing for independent competition. Every seven days, measurements were repeated and, thus, proportional changes of each population were tracked over time. The first measurement was used as a reference.

### 4.10. RT-qPCR

RNA-extraction was performed with the RNeasy Mini-Kit (Qiagen, Hilden, Germany), according to the manufacturer’s protocol. RNA concentration and purity were measured with the NanoDrop 2000 Spectrophotometer (ThermoFisher Scientific, Waltham, MA). For gene expression analysis, 1 µg of RNA was reverse-transcribed into cDNA using the QuantiTec Reverse Transcription Kit (Qiagen, Hilden, Germany). Each sample was run in triplicates with gene-specific primers (Hoxa9 Forward, 5′-CCC CGA CTT CAG TCC TTG C-3′; Hoxa9 Reverse, 5′-CCA GGA GCG CAT ATA CCT GC-3′; Meis1 Forward, 5′-TCC ACT CGT TCA GGA GGA AC-3′; Meis1 Reverse, 5′-TGC TGA CCG TCC ATT ACA AA-3′; Pla2g4a Forward, 5′-CAC CGC AAA GGT CTC ATT CCA CAC G-3′; Pla2g4a Reverse, 5′-AAA CCG TGT GGA ATG AGA CCT TTG C-3′) as well as with a β-actin reference (ß-Actin Forward, 5′-CCT CCC TGG AGA AGA GCT A-3′; ß-Actin Reverse 5′-TCC ATA CCC AAG AAG GAA GG-3′) using the QuantiTect Sybr Green PCR Kit (Qiagen, Hilden, Germany) and the Step One Plus Real-Time PCR System (Applied Biosystems, Foster City, CA, USA). Relative expression was calculated using the ΔΔCT method.

### 4.11. Dataset Analysis

For the identification of potential AML-related vulnerabilities, the GEO accession GSE75272 microarray dataset was downloaded and analyzed for differentially expressed genes between Hoxa9 (GSM1948614—17) and Hoxa9/Meis1 samples (GSM1948618—21) using the Transcriptome Analysis Console (TAC) software tool (ThermoFisher Scientific, Waltham, MA) [11]. Additionally, genes were uploaded to the GSEA software. Upregulated and leading edge-enriched genes were then individually investigated for their effect on survival using the cBioportal online tool and the TCGA-LAML dataset [26,64,65,66]. Disease-free survival, overall survival, and gene expression data were extracted from the same dataset using the UCSC Xena browser [67]. The final list of candidate genes was uploaded to the Genepattern HeatMapViewer tool for heat map generation. Bloodspot was used for dataset analysis of the normal human gene expression landscape compared to AML samples [41,68]. *PLA2G4A* expression was extracted from the following groups: AML t(15;17), AML inv [16]/t(16;16), AML t(8;21), AML complex, AML t(11q23)/MLL, and HSC. Furthermore, the UCSC Xena browser was used to extract *PLA2G4A* expression of MLLr AML and non-MLLr AML samples from the TARGET dataset of pediatric AML cases [67,69]. For functional protein association network analysis of human PLA2G4A the online software tool STRING was used [70]. Full STRING network analysis was performed. With active interaction sources based on text mining, experiments, databases, co-expression, gene neighborhood, gene fusion, and gene homology. Expression data of *HOXA9*, *MEIS1*, and *PLA2G4A* in MOLM13, THP1, Kasumi-1, OCI-AML3, MV-4-11, NOMO-1, and K562 cells were extracted from the Cancer Cell Line Encyclopedia (CCLE) using the Expression Atlas [37,38].

### 4.12. Statistical Analysis

All statistical analyses were performed with GraphPad Prism 8.3.0 (GraphPad Inc., San Diego, CA, USA). Kaplan–Meier survival curves were created to analyze overall survival (OS) and disease-free survival (DFS). Survival curve comparison was performed using log-rank tests. For appropriate univariate or multivariate analysis mean and standard deviation were calculated. Welch’s *t*-test or Student’s *t*-test were used for two-group comparison. Simple linear regression was performed to calculate the coefficient of determination (R2). Correlation was analyzed by Pearson’s correlation test. One-way ANOVA was used to determine statistical differences of three or more independent groups. Dunnett’s multiple comparison test was used to compare multiple groups with a single control. A *p*-value < 0.05 was considered to indicate a significant difference (* = *p* < 0.05; ** = *p* < 0.005; *** = *p* < 0.0005; **** = *p* < 0.0001).

## Data Availability

The results published here are in part based upon data generated by the Therapeutically Applicable Research to Generate Effective Treatments (https://ocg.cancer.gov/programs/target) (accessed on 15 September 2020) initiative, phs000218. The data used for this analysis are available at https://portal.gdc.cancer.gov/projects (accessed on 15 September 2020). The results published here are in part based upon data generated by the TCGA Research Network: https://www.cancer.gov/tcga (accessed on 15 September 2020).

## Supplementary Materials

The following are available online at https://www.mdpi.com/article/10.3390/ijms22179411/s1.
