# Supplementary material for: A Multiplex CRISPR-Screen Identifies PLA2G4A as Prognostic Marker and Druggable Target for HOXA9 and MEIS1 Dependent AML
Source: Int J Mol Sci. 2021 Aug 30;22(17):9411. doi: 10.3390/ijms22179411 (PMC8431012; doi:10.3390/ijms22179411)
Supplement: Supplementary file 1 [file ijms-22-09411-s001.zip › ijms-1252170-supplementary.pdf]

Supplementary Figure S1

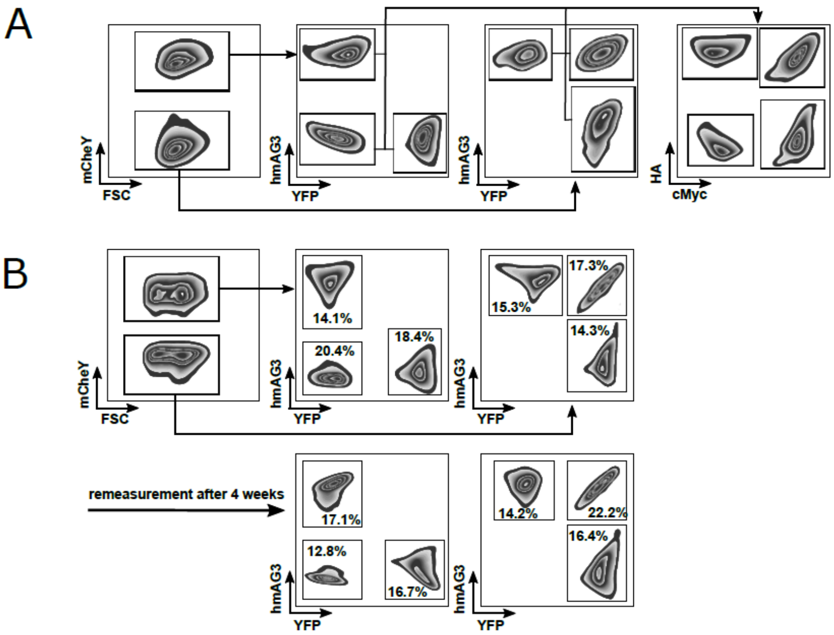

Figure S1: Gating strategies for the 6xFGB and 24xFGB vector system. (**A**) Gating strategy for 24xFGB multiplexing flow cytometry experiments. First cells are gated by mCheY expression. mCheY+ cells are further divided in mCheY+/ YFP+, mCheY+/ hmAG3+ or mCheY+ populations. mCheY- cells are divided in hmAG3+, YFP+ and hmAG3+/ YFP+ populations. Each of these six populations can be subdivided by surface marker expression HA and cMyc (HA+/cMyc+, HA+/ cMyc-, HA-, cMyc+, HA-, cMyc-) to give a total of 24 distinguishable populations. (**B**) Gating strategy for multiplex competitive proliferation assays using the 6xFGB vector system. First cells are gated by mCheY expression. mCheY+ cells are further divided in mCheY+/ YFP+, mCheY+/ hmAG3+ or mCheY+ populations. mCheY- cells are divided in hmAG3+, YFP+ and hmAG3+/ YFP+ populations. Each population size is determined by percentage of grandparent (total living cells). After a fixed timepoint the same sample is measured again. Comparing the relative population size changes over multiple timepoints allows for a detailed analysis of growth advantages or disadvantages of each population.

Supplementary Figure S2

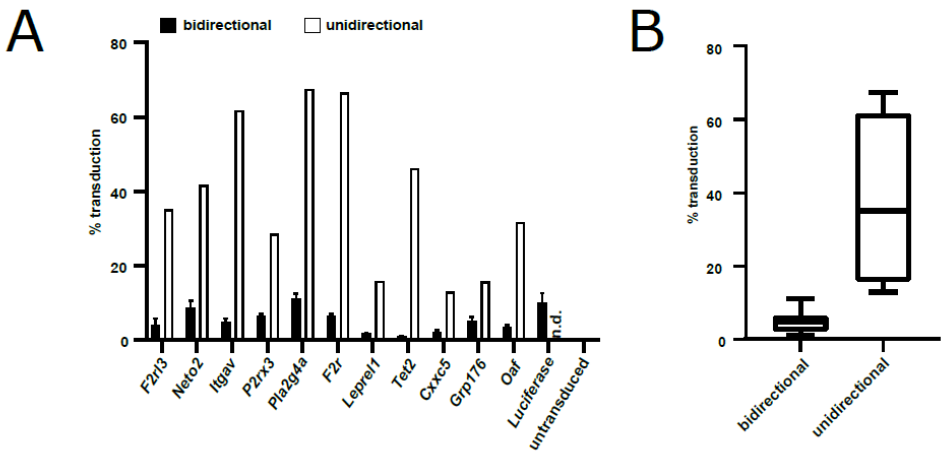

Figure S2: Comparison of transduction efficiencies. (**A**) Transduction efficiency comparison of unidirectional and bidirectional vectors. Bar graph shows direct comparison of corresponding vectors (mean ± SD, unidirectional n = 1, bidirectional n = 3). In addition to our 9 sgRNAs with recombination efficiencies >50%, we also included Gpr176 (recombination efficiency >40%) as well as sgRNAs against Tet2 and luciferase as positive and negative controls, respectively to obtain a total of 12 constructs. (**B**) Boxplot graph comparing pooled unidirectional and bidirectional vector transduction efficiency. The upper limit, center and lower limit of each box denotes the upper quartile, median and lower quartile, respectively. Whiskers representing min and max recombination samples.

Supplementary Figure S3

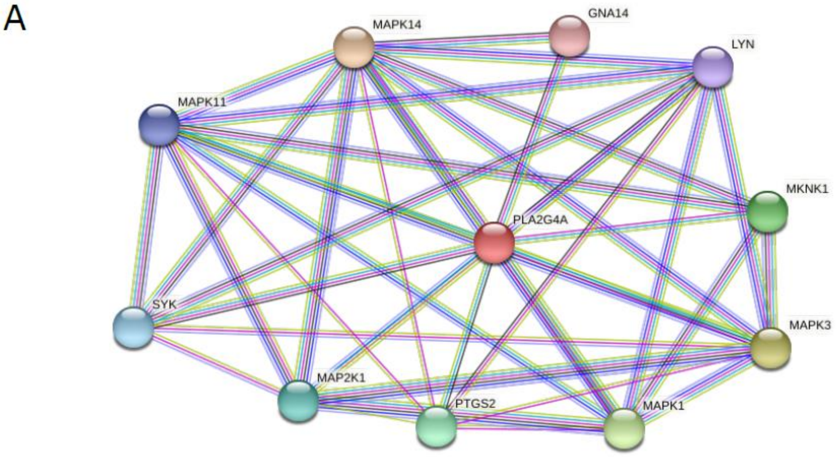

Figure S3: Protein-protein interaction networks of PLA2G4A. The string analysis depicts the ten most connected PLA2G4A interaction partners with prominent members of the MAPK family. Each string represents protein-protein associations of known or predicted interactions. The STRING score for predicted functional partners of PLA2G4A was calculated (MAPK14 = 0.971, MAPK3 = 0.969, MAPK1 = 0.968, MKNK1 0.951, PTGS2 = 0.946, MAP2K1 = 0.936, SYK = 0.934, MAPK11 = 0.928, LYN = 0.925, GNA14 = 0.923). The figure was generated with https://string-db.org/.

Supplementary Figure S4

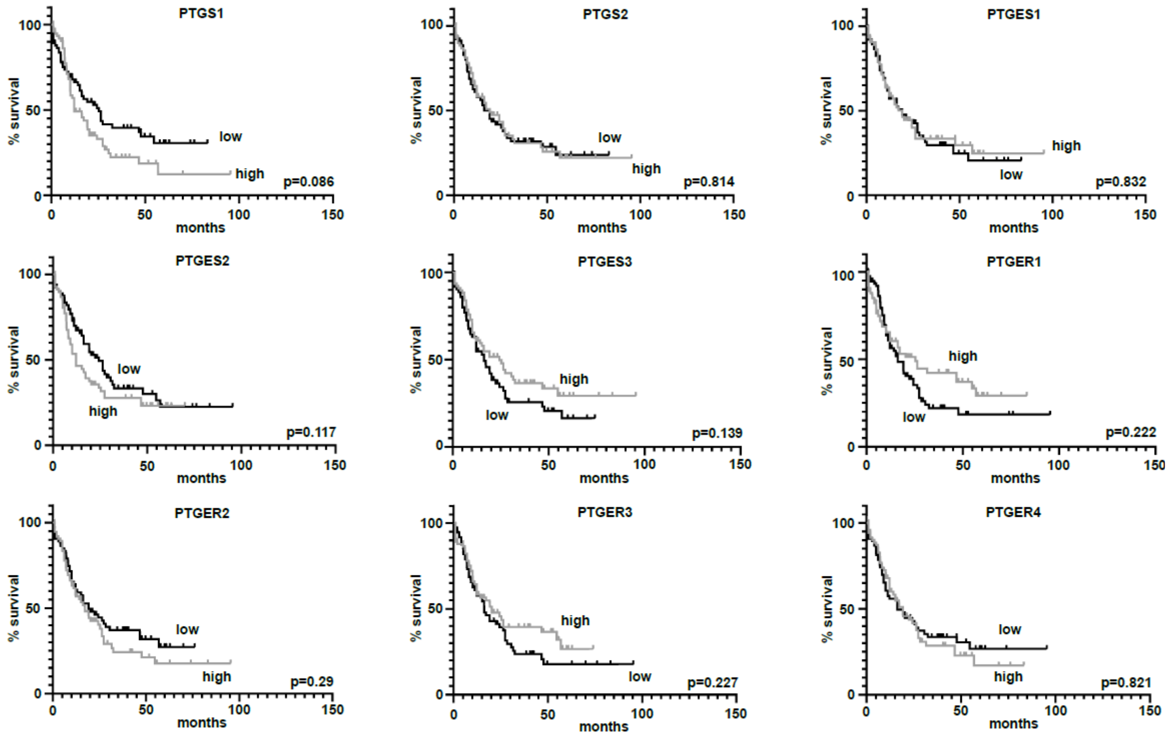

Figure S4: Prognostic potential of members of the PGE_2 synthesis and receptor pathway. Kaplan-Meier survival curve of overall survival in AML patients with above median gene expression compared to patients with below median gene expression (n = 121).

Supplementary Tables

Entrez. GeneID	Gene. Symbol	shRNA. ID	Primer_Name	Primer_Sequence
18783	Pla2g4a	Pla2g4a. 2357	Pla2g4a.2357_FW	AGCGAAAGCAAGAAAATTCTTCAATATAGTGAA GCCACAGATGTATATTGAAGAATTTTCTTGCTTC
			Pla2g4a.2357_REV	GGCAGAAGCAAGAAAATTCTTCAATATACATCT GTGGCTTCACTATATTGAAGAATTTTCTTGCTTT
18783	Pla2g4a	Pla2g4a. 1738	Pla2g4a.1738_FW	AGCGCCCAGATGAATTTGAACGAATATAGTGAA GCCACAGATGTATATTCGTTCAAATTCATCTGGA
			Pla2g4a.1738_REV	GGCATCCAGATGAATTTGAACGAATATACATCT GTGGCTTCACTATATTCGTTCAAATTCATCTGGG

Supplementary Table S1: shRNA-primer sequences. shRNAs were selected using the https://felixfadams.shinyapps.io/miRN/ online tool prior to the ligation of double stranded oligonucleotides into the lentiviral miR-N backbone.

**Gene**	**location**	**target site**	**hU6-FW / REV oligo**
Prdm5	Exon 1	GTTCGCCCTGAAGTCGTCCC**GGG**	CACCGTTCGCCCTGAAGTCGTCCC
			AAACGGGACGACTTCAGGGCGAAC
Cand2	Exon 2	GGATGAGGACAGCGAGCGTA**AGG**	CACCGGATGAGGACAGCGAGCGTA
			AAACTACGCTCGCTGTCCTCATCC
F2rl3	Exon 2	AATTTGCCCGGGTAGCCTCG**TGG**	CACCgAATTTGCCCGGGTAGCCTCG
			AAACCGAGGCTACCCGGGCAAATTC
F2r	Exon 1	GCGACGATCAGCAAGCGCCG**GGG**	CACCGCGACGATCAGCAAGCGCCG
			AAACCGGCGCTTGCTGATCGTCGC
Calcrl	Exon 7	TGCTGGAATGACGTTGCAGC**AGG**	CACCgTGCTGGAATGACGTTGCAGC
			AAACGCTGCAACGTCATTCCAGCAC
Cxxc5	Exon 2	CAGTTGATGCGCCGCCGGCA**GGG**	CACCgCAGTTGATGCGCCGCCGGCA
			AAACTGCCGGCGGCGCATCAACTGC
Oaf	Exon 3	TCAGCCTGCCGCACTGCGCG**GGG**	CACCgTCAGCCTGCCGCACTGCGCG
			AAACCGCGCAGTGCGGCAGGCTGAC
Itgav	Exon 2	GTGAATGCTCATCCAGCCGC**CGG**	CACCGTGAATGCTCATCCAGCCGC
			AAACGCGGCTGGATGAGCATTCAC
Pla2g4a	Exon 4	CAAAGGTCTCATTCCACACG**GGG**	CACCgCAAAGGTCTCATTCCACACG
			AAACCGTGTGGAATGAGACCTTTGC
Neto2	Exon 4	GTCAGCTCTATTCGTTGACG**AGG**	CACCGTCAGCTCTATTCGTTGACG
			AAACCGTCAACGAATAGAGCTGAC
P2rx3	Exon 2	GGCCGTGTCCCGCACTTGGT**AGG**	CACCGGCCGTGTCCCGCACTTGGT
			AAACACCAAGTGCGGGACACGGCC
Gucy1a3	Exon 5	CGAAGCGGGGATCACTAGCG**AGG**	CACCgCGAAGCGGGGATCACTAGCG
			AAACCGCTAGTGATCCCCGCTTCGC
Leprel1	Exon 3	GTATAAAGGTGGTCTTTACG**AGG**	CACCGTATAAAGGTGGTCTTTACG
			AAACCGTAAAGACCACCTTTATAC
Ednra	Exon 2	CACCCATCGACCCCCTAATT**TGG**	CACCgCACCCATCGACCCCCTAATT
			AAACAATTAGGGGGTCGATGGGTGC
Gpr176	Exon 1	GGCGCCAACCTCAGCGCGTT**CGG**	CACCGGCGCCAACCTCAGCGCGTT
			AAACAACGCGCTGAGGTTGGCGCC
Tet2 ctrl	Exon3	GAACAAGCTCTACATCCCGT**AGG**	CACCGAACAAGCTCTACATCCCGT
			AAACACGGGATGTAGAGCTTGTTC
Luciferase		AGTTCACCGGCGTCATCGTC**GGG**	CACCGAGTTCACCGGCGTCATCGTC
			AAACGACGATGACGCCGGTGAACTC

Supplementary Table S2: sgRNA target and primer sequences. Optimized sgRNAs were selected with the help of the CCTop (https://cctop.cos.uni-heidelberg.de:8043/) online tool. The table shows the location of each target site within the corresponding gene, the target site sequence including the protospacer adjacent motif (PAM; underlined and bold) as well as the two oligonucleotides for annealing and ligation together with a human U6 promoter fragment into the FGB vector vector backbone.
